# Cell-free DNA analysis reveals *POLR1D*-mediated resistance to bevacizumab in colorectal cancer

**DOI:** 10.1186/s13073-020-0719-6

**Published:** 2020-02-22

**Authors:** Qing Zhou, Samantha O. Perakis, Peter Ulz, Sumitra Mohan, Jakob M. Riedl, Emina Talakic, Sigurd Lax, Martin Tötsch, Gerald Hoefler, Thomas Bauernhofer, Martin Pichler, Armin Gerger, Jochen B. Geigl, Ellen Heitzer, Michael R. Speicher

**Affiliations:** 1 Institute of Human Genetics, Diagnostic and Research Center for Molecular Biomedicine, Medical University of Graz, Graz, Austria; 2 Present address: Freenome, South San Francisco, CA USA; 3 Present address: Cancer Research UK-Manchester Institute, Manchester, UK; 4 Department of Internal Medicine, Division of Oncology, Medical University of Graz, Graz, Austria; 5 Division of General Radiology, Medical University of Graz, Graz, Austria; 6 Department of Pathology, General Hospital Graz II, Graz, Austria; 7 Johannes Kepler University Linz, Linz, Austria; 8 Institute of Pathology, General Hospital Hochsteiermark, Leoben, Austria; 9 Institute of Pathology, Diagnostic and Research Center for Molecular Biomedicine, Medical University of Graz, Graz, Austria; 10 BioTechMed-Graz, Graz, Austria; 11 Christian Doppler Laboratory for Liquid Biopsies for Early Detection of Cancer, Graz, Austria

**Keywords:** Cell-free DNA, *POLR1D*, Bevacizumab, Whole-genome sequencing, Therapy resistance, Precision medicine

## Abstract

**Background:**

Bevacizumab, a monoclonal antibody against soluble VEGFA, is an approved and commonly administered anti-angiogenic drug in patients with metastasized colorectal cancer (mCRC). The survival benefit of anti-VEGF therapy in mCRC patients is limited to a few months, and acquired resistance mechanisms are largely unknown. Here, we employed whole-genome sequencing of plasma DNA to evaluate the tumor genome of patients undergoing treatment with bevacizumab to determine novel aberrations associated with resistance.

**Methods:**

Using longitudinal plasma analyses, we studied the evolution of tumor genomes in a mCRC cohort (*n* = 150) and conducted analyses of CRC cases from The Cancer Genome Atlas (TCGA) database (*n* = 619) to identify associations between genomic aberrations and clinical features. We employed whole-genome sequencing to identify the most frequently occurring focal somatic copy number alterations (SCNAs). Using the TCGA data as a comparative and supporting dataset, we defined the minimally amplified overlapping region and studied the mechanistic consequences of copy number gain of the involved genes in this segment. In addition, we established an in vitro cell model and conducted downstream gene expression and cell viability assays to confirm our findings from the patient dataset.

**Results:**

We observed a recurrent focal amplification (8.7% of cases) on chromosome 13q12.2. Analysis of CRC cases from the TCGA database suggested that this amplicon is associated with more advanced stages. We confirmed that this 13q12.2 amplicon frequently emerges later during the clinical course of disease. After defining the minimally amplified region, we observed that the amplification and expression of one gene, *POLR1D*, impacted cell proliferation and resulted in upregulation of *VEGFA*, an important regulator of angiogenesis which has been implicated in the resistance to bevacizumab treatment. In fact, in several patients, we observed the emergence of this 13q12.2 amplicon under bevacizumab treatment, which was invariably associated with therapy resistance.

**Conclusions:**

Non-invasive analyses of cell-free DNA from patients undergoing treatment with bevacizumab enabled the tracking of evolving tumor genomes and helped identify a recurrent focal SCNA of clinical relevance. Here, we describe a novel resistance mechanism against a widely applied treatment in patients with mCRC which will impact the clinical management of patients.

## Background

Colorectal cancer (CRC) still remains a large global health problem, representing the third most commonly diagnosed malignancy worldwide as well as one of the major causes of morbidity and mortality across populations [1]. Its burden is projected to increase by 60% by the year 2030 with an estimated 2.2 million new cases and 1.1 million deaths [2]. Reports have shown that almost 50% of individuals with CRC who are initially diagnosed to have localized cancer will subsequently develop metastases as the disease progresses [3], and furthermore, approximately 30% of patients present with distant metastases already at the time of diagnosis [4]. Although surgical removal of early-stage or metastatic lesions represents a potential curative approach [5], therapeutic options for metastatic CRC (mCRC) are commonly limited to palliative approaches that improve quality of life and survival for a median time of about 2 to 3 years. The identification of molecular targets and pathways involved in the initiation and progression of CRC have helped to better characterize the disease and further tailor patient treatment more precisely to minimize primary resistance, or to avoid it altogether [6]. Although advances in genome sequencing technology have enabled high-resolution detection of potential molecular targets, such as somatic copy number alterations (SCNAs) or mutations, the significance of many such aberrations remains elusive in terms of guiding therapy decision-making.

Liquid biopsies, i.e., the analysis of tumor components in bodily fluids such as blood [7–10], have shown promising clinical utility in the management of CRC, ranging from applications in early detection [11–17], detection of relapse [18–20], identification of prognostic markers [21, 22], molecular characterization of metastatic disease [23], and tracking response to therapy [24–28].

To this end, “driver” alterations, i.e., those that actively promote cancer development, are of particular importance [29]. Of particular relevance are focal SCNA events, i.e., aberrations spanning a restricted length of a chromosome arm, as these regions harbor a limited number of genes of which one or a few may give rise to a growth advantage as a result of selection during the evolution of the cancer genome [30–32]. Definitions of focal events somewhat vary throughout the literature. Therefore, we analyzed the SCNAs in the TCGA pan-cancer dataset and developed a very restrictive definition of an amplicon [33], the utility of which we were able to prove using low-coverage whole-genome sequencing of plasma DNA (plasma-Seq) [34] in several studies [35–38].

In the current study, we applied plasma-Seq to our mCRC cohort (*n* = 150). As compared to analysis of primary tumors, the analyses of plasma DNA offer the unique opportunity to establish the sequential order of events. As expected, we identified several focal SCNAs harboring known cancer driver genes, for example, chr12p12.1 and chr8p11.23-p11.22, which include *KRAS* and *FGFR1*, respectively. However, we also found recurrent focal amplifications in which cancer driver genes have not yet been established. The most frequent amplicon was on 13q12.2, which we detected in 14 patients, and our serial plasma analyses suggested that this amplicon is a rather late event and potentially associated with resistance to administered therapies. We assessed the biological role and clinical significance of this focal event in mCRC patients and, furthermore, investigated the functional role of the genes harbored within this limited chromosomal region.

## Methods

### Patient cohort

The study was approved by the Ethics Committee of the Medical University of Graz (approval number 21–229 ex 09/10), conducted according to the Declaration of Helsinki, and written informed consent was obtained from all patients.

The age and sex distribution of all patients is summarized in Additional file 1. All patients had metastatic CRC and were being treated at the Department of Internal Medicine, Division of Oncology, at the Medical University of Graz. We were able to isolate DNA from pre-treatment tumor specimens in nine patients for which tumor tissue was available as a result of surgical or bioptic procedures. Imaging studies, i.e., computed tomography (CT) scans, were obtained as part of routine clinical care.

As the focus of this study was on copy number profiling of ctDNA, which requires tumor fractions of 5% and higher, we pre-screened our plasma collection using our previously published modified Fast Aneuploidy Screening Test-Sequencing System [39] to identify appropriate plasma samples with elevated tumor content. Based on these mFAST-SeqS pre-screening results, the 150 patients included in this study were selected.

### TCGA data collection and analysis

TCGA data analyzed in this work originated from TCGA-COADREAD projects [40], and only cases with copy number variation data were kept. Clinical analyses and gene expression data were downloaded from Broad Institute GDAC Firehose (http://gdac.broadinstitute.org/). Absolute copy number results were downloaded from the NCI Genomic Data Commons (GDC; https://gdc.cancer.gov/about-data/publications/pancanatlas) [41]. Patient samples were categorized as “balanced,” “gain,” or “amplification” according to 13q12.2 copy number (balanced, 1 < copy number ≤ 3; gain, 3 < copy number ≤ 6; amplification, copy number > 6). Statistical analyses were performed in R. Focal event calling was performed by using an in-house script as published previously [35, 36].

### Definition of the minimally 13q12 amplified region and involved genes

We first determined the minimal overlapping range of all the focal events in our patient cohort (Table 1) by calculating the median log2 ratio of chr13 against all 14 patients who harbored the 13q12.2 SCNA of each pre-defined 50 kb window. For those windows which showed a median log2 ratio over 0.55, the frequency of focal events was counted (Additional file 2: Figure S3A). Using the windows that demonstrated the lowest frequency, we calculated a *p* value (Fisher’s exact test) in order to identify the statistically significant minimal overlapping range. We identified a broad peak (*p* < 0.05; Fisher’s exact test; chr13:27,708,804-28,667,235) and a focal peak (*p* < 0.01; Fisher’s exact test; chr13:28,441,650-28,667,235) in our patient dataset. To confirm this finding, we applied the same method to the TCGA dataset and ended up with a broad peak at chr13:28,197,436-28,650,763 (*p* < 0.05; Fisher’s exact test) and a focal peak at chr13:28,382,214-28,604,579 (*p* < 0.01; Fisher’s exact test), which is comparable with the GISTIC analysis result (broad peak: chr13:28,192,985-28,773,237; focal peak: chr13: 28,391,954-28,558,679) (Additional file 2: Figure S3A).

**Table 1:** Summary of clinical information of all cases harboring 13q12.2 focal amplification

Patient ID	Disease stage (TC)	Disease stage (PC)	Location	cM (TC)	cM (PC)	TBC (months)	Detection*
C74	IV B	IV B	Rectum	M1	M1	22.8	Yes
C79	NA	NA	NA	NA	NA	NA	No
C95	II A	IV B	Left flexure	M0	M1	43.4	NA
C109	III B	IV B	Rectum	M0	M1	101.7	Yes
C110	IV A	IV B	Colon ascending	M1	M1	0.7	NA
C118	IV B	IV B	Colon sigmoid	M0	M1	22.9	No
C112	III A	IV B	Rectum	NA	NA	27.1	NA
C123	III B	IV B	Colon sigmoid	M0	M1	76.5	Yes
C129	I	IV B	Colon sigmoid	M0	M1	121.3	NA
C166	IV A	IV A	Colon transverse	M1	M1	0.7	No
C178	III C	IV B	Cecum	M0	M1	5.4	Yes
C206	III B	III B	Rectum	M0	M0	7.6	No
C240	III B	IV B	Colon sigmoid	M0	M1	79.9	No
C216+	NA	IV B	NA	NA	NA	NA	NA

The age range of the 14 patients was 45–81 years (mean = 60.7 years, median = 51 years), 36% were female, and 64% were male. *TC* tissue collection, *PC* plasma collection, *TBC* time to first blood collection after diagnosis

*13q12.2 SCNA found in primary tissue; +13q12.2 SCNA was not detected in the first blood draw in patient C216

### Plasma-seq: whole-genome sequencing of primary tumor and plasma samples

Whole-genome sequencing libraries were prepared and sequenced for plasma and tumor samples when available by methods described previously in detail [34, 36, 42]. In brief, plasma DNA was isolated using the QIAamp Circulating Nucleic Acid Kit (Qiagen, Hilden, Germany) from 1 to 2 mL of plasma and primary tumor DNA was isolated from FFPE using the GeneRead DNA FFPE kit (Qiagen, Hilden, Germany). Samples were quantified with the Qubit dsDNA HS Assay Kit (Thermo Fisher Scientific, Vienna, Austria). Shotgun libraries were prepared using the TruSeq DNA LT Sample preparation Kit (Illumina, San Diego, CA, USA) according to the manufacturer’s instructions for both primary tumor samples and for cell lines, but with several modifications for the generation of plasma libraries: 5–10 ng of input DNA was used and the fragmentation step was omitted, since plasma DNA is enriched for fragments in the range of 160 to 340 bp, and 25 PCR cycles were used for the selective amplification step of library fragments. Libraries were sequenced on either an Illumina MiSeq or NextSeq 550 instrument (Illumina, San Diego, CA, USA) for the generation of 150 bp single reads or 76 bp paired end with 5–10 million reads per sample, representing a 0.1–0.2× coverage of the whole genome. SCNA data analysis was performed as described previously [34]. Tumor fraction from plasma DNA and tumor samples were estimated with the ichorCNA algorithm, a probabilistic model for the simultaneous prediction of large-scale copy number alterations and estimation of tumor fraction, which is equivalent to tumor purity from bulk tumor analyses [43].

### Digital PCR copy number assay

SCNAs of *POLR1D* and *ERBB2* were analyzed using digital PCR (dPCR) and performed on the QuantStudio 3D platform (Life Technologies, Carlsbad, CA, USA). Pre-designed TaqMan assays specific for the detection of the copy number of *POLR1D* (Hs02926936_cn), *ERBB2* (Hs00450668_cn), and a reference assay (*TERT*; 4403315) were purchased from Thermo Fisher. For dPCR, a total amount of 3–5 ng plasma DNA was used as input and samples were run in duplicate using the QuantStudio™ 3D Digital PCR 20 K Chip Kit v2 and a QuantStudio 3D instrument (Life Technologies, Carlsbad, CA, USA). Raw data were analyzed using the relative quantification module of the QuantStudio 3D Analysis Suite Software, including a Poisson correction. The confidence level was set to 95%, and the desired precision value was 10%.

### Cell lines and cell culture

The human CRC cell lines OXCO-2, SW480, and HT29 were selected based on their copy number variation profiles and transfection suitability as in vitro cell models. OXCO-2 was kindly provided by Dr. Alberto Bardelli, Laboratory Molecular Oncology at the Candiolo Cancer Institute IRCCS-Candiolo (Torino). SW480 and HT29 cell lines were provided by Prof. Martin Pichler, Department of Internal Medicine, Division of Oncology, Medical University of Graz. Cell line SCNAs were profiled using whole-genome sequencing as described previously. SW480 cells harbor a complete gain of chromosome 13, whereas HT29 harbors a focal amplification of chr13q12.2. OXCO-2 cells have no changes in chromosome 13 (Additional file 2: Figure S2).

OXCO-2 cells were maintained in Iscove’s modified Dulbecco’s medium (IMDM; Gibco, Thermo Fisher Scientific, Vienna, Austria). HT29 cells were maintained in Dulbecco’s modified Eagle’s medium (DEME; Gibco, Thermo Fisher Scientific, Vienna, Austria), and SW480 cells were maintained in RPMI 1640 Medium (Gibco, Thermo Fisher Scientific, Vienna, Austria). All media were supplemented with 5% fetal bovine serum (Gibco, Thermo Fisher Scientific, Vienna, Austria) and 1% penicillin/streptomycin (Gibco, Thermo Fisher Scientific, Vienna, Austria). The CRC cell lines were authenticated at the Cell Bank of the Core Facility of the Medical University of Graz, Austria, by performing a STR profiling analysis (Kit: Promega, PowerPlex 16HS System; cat. no. DC2101, last date of testing: July 2019).

### Generation of stable FLT3-overexpressing cell line

OXCO-2 cells were seeded in a 6-well plate in IMDM media at approximately 80% confluence and incubated overnight. The FLT3 (NM_004119) GFP-tagged human cDNA ORF clone (Acris, Herford, Germany) was transfected into cells 24 h post-seeding using the FuGene HD (Promega, Mannheim, Germany) transfection reagent according to the manufacturer’s recommendations. Cells were cultured in IMDM media containing 1 mg/mL Geneticin (Gibco, Thermo Fisher Scientific, Vienna, Austria) starting 24 h after transfection.

Colonies were collected via cloning discs (Sigma-Aldrich/Merck KGaA, Darmstadt, Germany) dipped in trypsin, and once detached, each colony was transferred separately into a 96-well plate containing IMDM media and Geneticin. Colonies originating from the single clones were expanded gradually into 6-well plates once they reached sufficient confluence with continued Geneticin treatment to further select for clones stably expressing FLT3-GFP (OXCO2-FLT3-GFP).

### siRNA knockdown assays

Pre-designed siRNAs (Additional file 3: Table S3; Ambion, Life Technologies) were reverse transfected into SW480 and HT29 cells using Lipofectamine™ RNAiMAX transfection reagent (Invitrogen, Thermo Fisher Scientific, Vienna, Austria) as suggested by the supplier. Transfected cells were incubated for 72 h before performing proliferation assay or harvesting for expression analysis.

### RNA isolation, quantitative RT-PCR, and mRNA-seq

RNA was isolated via the TRIzol (Invitrogen, Thermo Fisher Scientific, Vienna, Austria) method and reverse transcribed into cDNA using the Omniscript RT Kit (Qiagen, Hilden, Germany). Equal amounts of RNA were used in cDNA synthesis. Quantitative RT-PCR was performed on the ABI 7500 system using Biozym Blue S’Green qPCR Kit (Biozym, Hessisch Oldendorf, Germany) and pre-designed RT-PCR primers (Table 2; Microsynth AG, Switzerland) according to the manufacturer’s suggestions.

**Table 2:** Summary of all RT-PCR primers and PCR protocols used

Gene	Forward primer sequence (5′ → 3′)	Reverse primer sequence (5′ → 3′)	PCR protocol	Cycles
FLT3	TTTCACAGGACTTGGACAGAGATTT	GAGTCCGGGTGTATCTGAACTTCT	95 °C/50 °C/72 °C	40
VIM	GAACTTTGCCGTTGAAGCTG	TCTCAATGTCAAGGGCCATC	95 °C/56 °C/73 °C	40
CDH1	GTCAGGTGCCTGAGAACG	GCCATCGTTGTTCACTGG	95 °C/56 °C/73 °C	40
POLR1D (V1)	CCACCTGAGGATCCAGAAAC	CCTCGTGCAATACAAATGTCA	95 °C/60 °C	40
POLR1D (V2)	AAGAACTGCTTAAGGAGGCAA	TCTTCGCTGGTTCCTTATCG	95 °C/60 °C	40
PAN3	TTGGTGCCCTCAACATCTCT	TTGATCCCATCGGAACTAGC	95 °C/60 °C	40
CDX2	GAACCTGTGCGAGTGGATG	TCCTCCGGATGGTGATGTAG	95 °C/60 °C	40
PDX1	CCTTTCCCATGGATGAAGTC	TTCAACATGACAGCCAGCTC	95 °C/60 °C	40
LNX2	ATGCAACGTTGTGATCTGGA	CCAAACAGTCTGCTTCTGGA	95 °C/60 °C	40
GAPDH	CCAAAATCAAGTGGGGCGATG	AAAGGTGGAGGAGTGGGTGTCG	95 °C/60 °C	40

Captured coding transcriptome RNA-seq libraries were prepared using the TruSeq RNA Exome kit (Illumina, San Diego, CA, USA) according to the manufacturer’s instructions with 100 ng total RNA input. Libraries were quantified using the Agilent High Sensitivity DNA Kit (Agilent Technologies, Santa Clara, CA, USA) and sequenced on an Illumina NextSeq instrument (Illumina, San Diego, CA, USA) for the generation of 75 bp paired-end reads. A pseudo-alignment approach (kallisto) was used to analyze RNA-seq data [44]. The output data of kallisto was reformed, and differential gene expression analysis was performed in R using the DESeq2 Bioconductor package [45].

### Colony formation and cell viability assay

For the colony formation assay, cells were seeded in 24-well plates for 72 h and fixed in 100% methanol for 20 min. Following staining with 0.5% crystal violet, images of each well were acquired. Colonies were counted using ImageJ software.

Cell viability was assessed with the CellTiter-Glo Luminescent Cell Viability Assay (Promega, Mannheim, Germany) according to the manufacturer’s instructions.

### Gene expression prediction

Gene expression prediction analysis was performed as described previously [38]. In brief, whole-genome libraries were sequenced to obtain 200 million reads (5× coverage) and coverage values around transcription start sites (TSSs) were extracted from aligned BAM files and normalized by the mean value of the combined regions: TSS − 3000 to TSS − 1000 and TSS + 1000 and TSS + 3000. The coverage from − 1000 to + 1000 bp (2K-TSS coverage) and from − 150 to + 50 bp (NDR coverage) was used for the identification and prediction of genes as expressed or unexpressed by using support vector machines (SVMs). The SVM used for these analyses was trained on transcription start sites of genes that are constitutively expressed and constitutively unexpressed within the same sample. For every sample, a separate model is trained based on a *k*-fold cross-validation on those samples and the remaining genes are predicted from the trained model within each fold. Only genes that are consistently predicted across folds (the same prediction in > 95% of folds) are considered to be valid predictions. We did not use external training data to avoid inconsistencies in the models.

## Results

### Recurrent focal events identified by plasma-Seq

**Fig. 1:**
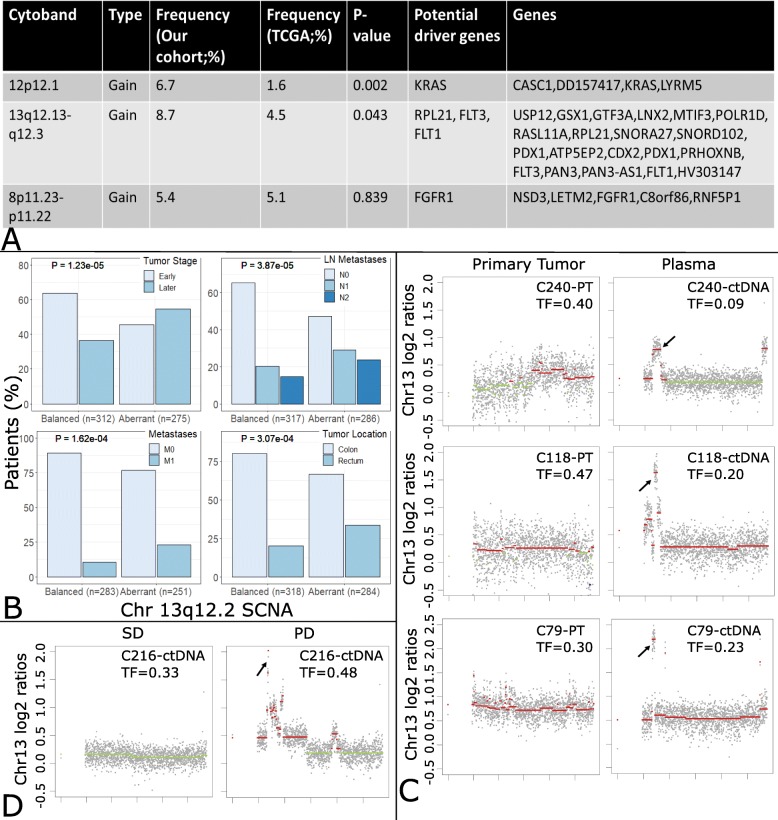
Identification of the 13q amplicon and establishment as a late event in CRC. **a** Recurrent focal events from our patient cohort with a frequency higher than 5%. Potential driver genes were identified according to a machine learning-based method for driver gene prediction [46]. The difference in these 3 recurrent focal events between our cohort and the TCGA cohort was analyzed using the chi-squared test. **b** The TCGA cohort was separated into 2 groups, i.e., balanced and aberrant (including gain and amplification cases). Bar charts illustrate 4 clinical features, i.e., tumor stage, distant metastasis, lymph node metastasis, and tumor location, which showed significant differences between these 2 groups. *p* values were calculated using the chi-squared test. **c**, **d** Plots illustrating the log2 ratio changes on chromosome 13. In C240, C118, and C79, focal amplification of chr13q12.2 was not identified in the primary tumor (PT) but found in the plasma (ctDNA) at a later stage. In C216, chr13q12.2 amplification was detected when the patient status was categorized as progressive disease. Copy number gains are shown in red and balanced regions in green. Tumor fraction (TF) of every sample was calculated using ichorCNA [43]. (SD, stable disease; PD, progressive disease)

Using plasma-Seq and our aforementioned criteria for focal SCNAs [33, 35], we identified several recurrent focal events in a set of 150 mCRC patients (Additional file 1), 3 of which were found to be present in more than 5% of patients, comprising 12p12.1, 13q12.13-q12.3, and 8p11.23-p11.22 (Fig. 1a, Additional file 2: Figure S1A, Additional file 4). To compare our data with other large-scale analyses, we used publicly available SCNA data from 619 CRC patients included in The Cancer Genome Atlas (TCGA), of which a minority (*n* = 85) are mCRC patients. We found many similarities and few differences in focal SNCAs. Some amplifications containing known driver genes, e.g., *ERBB2*, *EGFR*, and *MYC*, and the deletion of tumor suppressor genes, e.g., *CDKN2A* and *MAP2K4*, were identified at a similar percentage in both cohorts (Additional file 4). The focal amplification in 8p11.23-p11.22 was also detected in both cohorts at a similar percentage (Fig. 1a, Additional file 2: Figure S1A, Additional file 4). In contrast, the focal amplification 12p12.1 harboring the *KRAS* oncogene was present in 6.7% of our patients, compared to only 1.6% of patient of the TCGA cohort (*p* = 0.002; chi-square test; Fig. 1a, Additional file 2: Figure S1A, Additional file 4), which may reflect a higher number of patients who received anti-EGFR therapy in our cohort [28, 36]. Moreover, the recurrent focal amplification of 13q12.13-q12.3 was observed with a significantly higher frequency in our cohort (8.7% vs 4.5%; *p* = 0.043; chi-square test; Fig. 1a, Additional file 2: Figure S1A, Additional file 4).

With the exception of the 13q12 amplification, known candidate driver genes were located in all other recurrent focal amplifications. Because of the high frequency of this amplicon in our cohort, we therefore focused on a more detailed characterization of this region.

### The 13q12.13-12.3 amplicon is associated with late-stage clinical features

As we only analyzed mCRC patients, whereas the TCGA data is mainly comprised of primary tumors, 55% of which are localized stages (I and II), the 13q12.13-12.3 amplicon may be more related to late-stage events of CRC. Indeed, in the TCGA dataset, patients with this 13q12 SCNA represented more late-stage disease (stage III and stage IV) (*p* = 1.23E−05; chi-square test), with distant (*p* = 1.62E−04; chi-square test) and lymph node metastases (*p* = 3.87E−05; chi-square test) (Fig. 1b). Furthermore, the 13q12 amplified cohort was significantly associated with primary tumors located in the rectum when compared to patients without the SCNA (*p* = 3.07E−04; chi-square test) (Fig. 1b).

We had access to the corresponding primary tumor in 9 of 14 patients with the amplification. Copy number analyses of the corresponding FFPE tumor tissue samples revealed that a focal amplification was already present in the primary tumor tissue in 4 patients (C123, C109, C178, and C74), whereas in 5 patients (C240, C118, C79, C206, and C166), this focal amplification was acquired at a later time point (Table 1, Fig. 1c, Additional file 2: Figure S1B and S2). In 3 patients with an undetected amplification in the primary tumor tissue (C240, C118, C79), the 13q12.13-q12.3 focal amplification appeared in 1 of the plasma DNA analyses after metastases were detected or the patient developed progressive disease (Table 1, Fig. 1c, Additional file 2: Figure S1B and S2). Furthermore, in 1 additional patient (C216), from whom an FFPE sample was not accessible, the amplification was not present in the first plasma sample but rather acquired after the patient exhibited progressive disease (Fig. 1d, Additional file 2: Figure S2). In 2 patients (C74, C123) with a detected gain in the primary tumor tissue, the copy number of the 13q12.13-q12.3 amplification increased in relation to the tumor fraction once metastases were acquired or progressive disease was exhibited (Table 1, Additional file 2: Figure S1B and S2), an observation additionally confirmed by dPCR.

### The oncogene *FLT3* is not associated with proliferation of CRC cells

The aforementioned data suggested that the 13q12.2 amplicon is associated with late-stage and progressive disease. In order to identify the potential driver in the amplicon, we first determined the minimal overlapping range of all the focal events in our patient cohort (Additional file 2: Figure S3A). For further confirmation, we applied the same method to the TCGA dataset and identified a broad and a focal peak, which were comparable with the GISTIC analysis result (Additional file 2: Figure S3A; further details of this region definition in the “Methods” section). In total, seven genes (*POLR1D*, *GSX1*, *PDX1*, *ATP5EP2*, *CDX2*, *PRHOXNB*, and *FLT3)* were completely located within the broad peak for all three analyses (Additional file 2: Figure S3A).

**Fig. 2:**
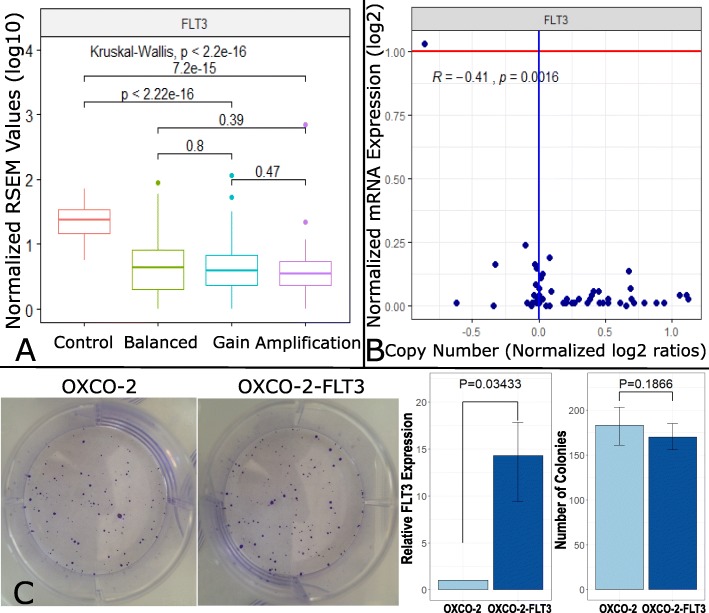
Exclusion of *FLT3* as a driver gene. **a** Box plot showing no significant correlation between *FLT3* gene copy number and FLT3 mRNA expression (log10 (normalized RSEM value + 1)) in the TCGA cohort. Control/matched normal tissue, *n* = 51; Balanced, *n* = 196; Gain, *n* = 129; Amplification, *n* = 46. **b** The scatter plot illustrates no correlation in *FLT3* copy number and FLT3 mRNA expression (log2 (TPM + 1)) in 58 CRC cell lines (*R* = − 0.41, *p* = 0.0016; Pearson). The red line represents the noise threshold (TPM = 1). **c** Colony formation assay showing significant overexpression of FLT3 in OXCO-2 cells (*p* = 0.03433; *t* test) but no significant changes in proliferation (*p* = 0.1866; *t* test)

Since *FLT3* is a well-known driver gene in hematological malignancies that can be targeted by the drug sorafenib [47], *FLT3* was considered to be a reasonable driver candidate. In order to understand the potential oncogenic role of *FLT3* in CRC, we investigated whether the *FLT3* gene amplification correlated with gene expression in the TCGA and the CCLE CRC cell line datasets, respectively. However, consistent with a previous report, we did not observe a correlation between mRNA expression and copy number of *FLT3* (Fig. 2a, b) [48]. To confirm this observation in vitro, we generated a CRC cell line stably expressing *FLT3*. As expected, overexpression of *FLT3* in OXCO-2 cells did not lead to a significant change in cell proliferation (Fig. 2c, *p* > 0.05; *t* test).

### Identification of *POLR1D* as a potential driver gene in 13q12.2

As the abovementioned results suggested that *FLT3* may not function as a driver gene in CRC, we investigated the other five candidate genes (pseudogene *ATP5EP2* was excluded) located in the overlapping broad peak regions as well as the first immediate upstream and downstream genes, *LNX2* and *PAN3*, respectively.

**Fig. 3:**
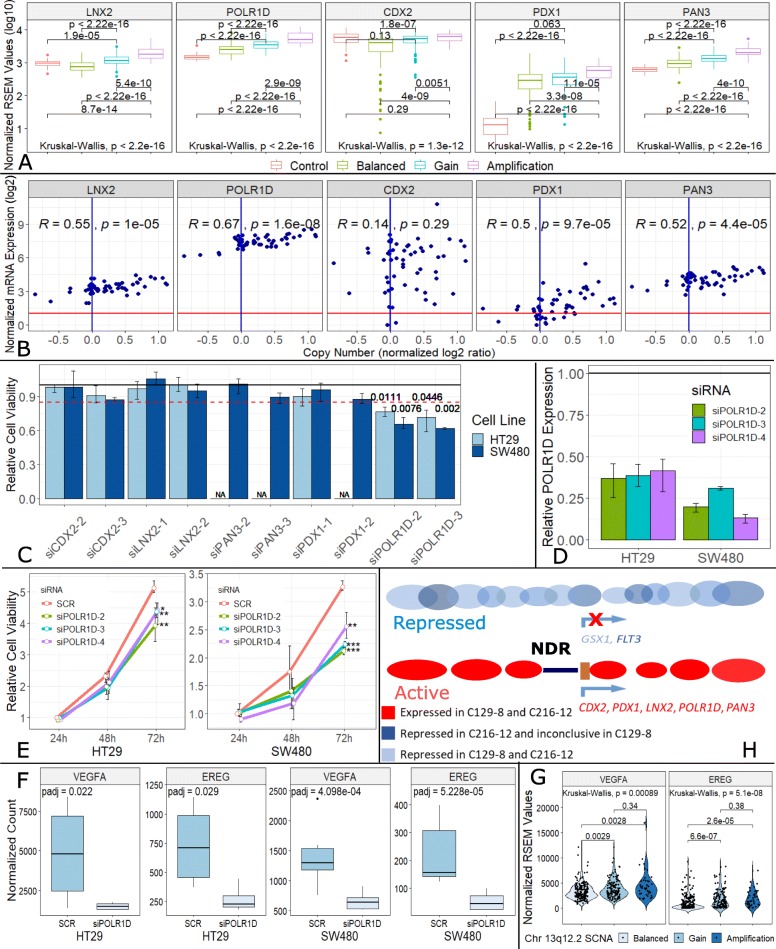
Expression analyses for identification of the potential driver gene in the 13q12.2 amplicon. **a** Box plots showing significant positive correlation between gene copy number and mRNA expression (log10 (normalized RSEM value + 1)) in 5 genes (i.e., *LNX2*, *POLR1D*, *CDX2*, *PDX1*, and *PAN3*) in the TCGA cohort. Control/matched normal tissue, *n* = 51; Balanced, *n* = 196; Gain, *n* = 129; Amplification, *n* = 46. **b** Scatter plots illustrating positive correlation in gene copy number and mRNA expression (log2 (TPM + 1)) in 5 genes (i.e., *LNX2*, *POLR1D*, *CDX2*, *PDX1*, and *PAN3*) in 58 CRC cell lines. *R* values and *p* values were calculated using Pearson’s correlation test. The red line represents the noise threshold (TPM = 1). **c** The bar chart illustrates cell viability changes after knockdown of 5 genes (i.e., *CDX2*, *LNX2*, *PAN3*, *PDX1*, and *POLR1D*) in 2 CRC cell lines (i.e., HT29 and SW480). Silencing of *POLR1D* in both cell lines showed reduction in cell viability over 15%. *p* values calculated by *t* test are shown above the bar. **d** Silencing of *POLR1D* with 3 different siRNA constructs. RT-PCR showing that silencing provided sufficient knockdown of POLR1D expression in both cell lines. **e** Cell viability time curve illustrating significant reduction of cell viability after knockdown of POLR1D expression in HT29 and SW480 cells (*p < 0.1; **p < 0.05; ***p < 0.01; *t* test). **f** Box plot illustrating the different expression (normalized DESeq2 read count) of VEGFA and EREG between negative control (SCR, a scrambled siRNA) and POLR1D knockdown in SW480 (SCR, *n* = 6; siPOLR1D2, *n* = 3; siPOLR1D3, *n* = 3) and HT29 (SCR, *n* = 4; siPOLR1D2, *n* = 2; siPOLR1D3, *n* = 2) cell lines. VEGFA and EREG expression was suppressed after POLR1D silencing. Adjusted *p* values were calculated by DESeq2, an R package. **g** Violin plots of VEGFA and EREG expression (normalized RSEM value) of TCGA cases. Samples with chr13q12.2 gain (*n* = 129) or amplification (*n* = 46) showed a significant upregulation compared to balanced cases (*n* = 196). **h** Schematic presentation how nucleosome organization around promoters of repressed and active genes differ in their promoter regions. Promoters of active genes have a nucleosome-depleted region (NDR, dark blue line), whereas the nucleosome organization of promoters of repressed genes is not well-defined, resulting in different nucleosome footprints at transcription start sites. We leveraged these differences by employing our previously published nucleosome positioning [38] to determine the expression status of genes within the 13q12.2 amplicon. In addition to the genes discussed in the text, we added the gene *GSX1* (light blue) as an example for a repressed gene (part of the figure adapted from [49])

In five of these genes, including *LNX2*, *POLR1D*, *CDX2*, *PDX1*, and *PAN3*, a positive correlation between copy number and mRNA expression could be demonstrated using the publicly available datasets from the TCGA and the CCLE databases (Fig. 3a, b; Additional file 2: Figure S3B and C). To further characterize a potential involvement of these genes in vitro, we induced a transient siRNA knockdown of these five genes in the two CRC cell lines HT29 and SW480, where 13q12.2 is overrepresented either due to a focal amplification (HT29) or gain of the entire chromosome 13 (SW480) (Additional file 2: Figure S2) and where these genes are expressed. Intriguingly, only silencing of *POLR1D* but not of the other genes demonstrated a significant reduction (1.3–1.6-fold) in cell viability in both the HT29 and SW480 cell line culture systems (Fig. 3c–e, Additional file 2: Figure S4A). These results suggest that *POLR1D* may have functional implications for CRC cell proliferation. *POLR1D* is a subunit of both RNA polymerases I and III. RNA polymerase I is involved in the production of 18S, 5.8S, and 28S rRNAs, while RNA polymerase III synthesizes small RNAs [50]. Despite a recent report describing the frequent overexpression of *POLR1D* in CRC [51], a role of *POLR1D* in cancer has not otherwise been thoroughly described in the literature.

### *POLR1D* affects expression of VEGFA and EREG

We sought to elucidate the underlying oncogenic mechanisms of *POLR1D* using RNA-seq analysis. In addition to *POLR1D*, which showed an approximate 2-fold reduction in expression in the silenced cells, we detected 44 differentially expressed genes in both the HT29-POLR1D-silenced and SW480-POLR1D-silenced cell lines compared to the controls.

Moreover, a similar expression change of 8 of these 45 total genes including *POLR1D* (i.e. *PPP1R15A*, *MOSPD2*, *FAM84B*, *GARS*, *POLR1D*, *KIF21B*, *VEGFA*, and *EREG*) was also observed in the TCGA cases with a 13q12 SCNA (Fig. 3f, g, Additional file 2: Figure S4B, Additional file 3: Table S1). All 45 genes demonstrated an upregulation in expression in patients harboring the 13q12 SCNA compared to patients with a balanced 13q12 region (Additional file 3: Table S1).

Of particular relevance appeared to be the POLR1D-associated upregulation of VEGFA and EREG. *VEGFA* encodes vascular endothelial growth factor A (VEGFA), which is an important regulator of angiogenesis in the PDGF/VEGF growth factor family and which plays a role in the development and progression of CRC [52]. The anti-VEGF monoclonal antibody bevacizumab (Avastin) was approved by the US Food and Drug Administration (FDA) for the treatment of advanced CRC [53] and is a recombinant humanized IgG1 antibody against all isoforms of VEGFA. Anti-VEGF treatment may induce the expression of VEGFA, which, in turn, is involved in the resistance to anti-VEGF treatment [54, 55]. *EREG* encodes epiregulin, which is a member of the epidermal growth factor (EGF) family. Epiregulin is a ligand of the epidermal growth factor receptor (EGFR) and the structurally related erb-b2 receptor tyrosine kinase 4 (ERBB4) [56]. Epiregulin promotes the progression of various cancers [57, 58].

### Nucleosome positioning mapping to infer *POLR1D* expression in plasma

Previously, we had shown that serial monitoring of tumor genomes by plasma DNA analyses may reveal focal amplifications as a mechanism of resistance to administered therapies in CRC [36] and in prostate cancer [35]. As we reasoned that due to the higher expression of VEGFA, tumors with a 13q12.2 amplification might be more resistant to anti-VEGF treatment, we implemented this serial monitoring concept here and investigated longitudinal plasma samples from two patients, i.e., C216 and C129, where we observed the emergence of the 13q12.2 amplicon under anti-VEGF treatment as described in detail below.

However, the observation of a novel amplification does not allow the conclusion that the genes located within the amplified region are actually expressed. cfDNA consists predominantly of nucleosome-protected DNA shed into the bloodstream by cells undergoing apoptosis [11, 59]. Transcription start sites (TSSs) harbor distinct nucleosome footprints, which differ for expressed and silent genes (Fig. 3h) [49]. Recently, we showed that after high-coverage whole-genome sequencing of plasma DNA, these TSS-nucleosome occupancy patterns can be leveraged to infer which cancer driver genes in regions with somatic copy number gains are expressed with high accuracy [38]. Hence, we selected for each of these two patient cases one plasma sample (C129-8 and C216-12), which both had the focal amplification on 13q12.2, and generated whole-genome libraries with 200 million reads (5× coverage) to conduct our previously described TSS profiling analyses [38].

These analyses predicted expression of *POLR1D* in both plasma samples along with the other four genes (*LNX2*, *CDX2*, *PDX1*, and *PAN3*) for which we had established a positive correlation between copy number and mRNA expression (Fig. 3h). In contrast, *FLT3* was classified as unexpressed in C216-12 and inconclusive in C129-8 (Fig. 3h). Hence, the gene expression inference from our nucleosome positioning mapping approach suggested that our observations from the cell culture systems are applicable to these clinical cases and, furthermore, that *FLT3* is indeed not the cancer driver gene within this amplicon in CRC.

### The emergence of *POLR1D* amplification correlates with resistance to bevacizumab

**Fig. 4:**
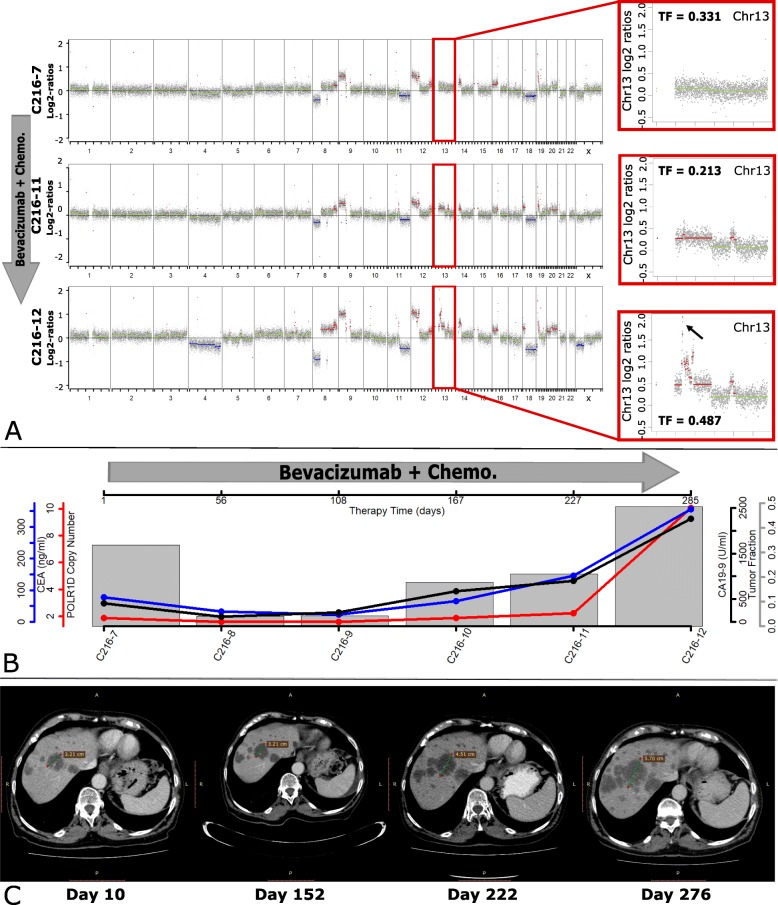
Emergence of the 13q12 amplicon under bevacizumab treatment in patient C216. **a** Genome-wide log2 ratio plots of plasma samples from C216 obtained before bevacizumab treatment (upper), after 227 days of bevacizumab treatment (middle), and after 285 days of bevacizumab treatment (bottom). The insets illustrate the respective tumor fraction (TF) for each analysis and enlarged log2 ratio plots of chromosome 13, the bottom 2 samples show gain of chromosome 13, with the highest copy number gain on chr13q12.2, the region that harbors the *POLR1D* gene. Copy number gains are shown in red, balanced regions in green, and copy number losses in blue. **b** Plot illustrating all time points of blood collection and relative marker changes. Red line: *POLR1D* copy number changes identified by digital PCR, showing minimum changes until day 227. Blue line: blood CEA level changes. Black line: blood CA19-9 level changes. Gray bar: tumor content identified in every sample using ichorCNA. **c** Four CT images obtained in 4 different time points, i.e., day 10, day 152, day 222, and day 276 after bevacizumab treatment. Compared to the first image, no significant changes were identified on day 152, during which the patient had been evaluated to have stable disease in accordance with the RECIST criteria. On day 222, the pre-present liver metastasis lesions enlarged with occurrence of new micrometastasis lesions. On day 276, all livers metastasis lesions had become larger

In patient C216, who had been undergoing anti-VEGF treatment for 9 months, we detected the emergence of the 13q12.2 amplification which correlated with the development of progressive disease and resistance to bevacizumab, i.e., increase in blood CEA and CA19-9 tumor markers and increase in lesion size of liver metastases, as observed in the CT scan (Fig. 4; Additional file 3: Table S2; Additional file 2: Figure S5). Digital PCR was used on all serial samples to confirm the emergence of *POLR1D* under anti-VEGF therapy (Fig. 4b).

**Fig. 5:**
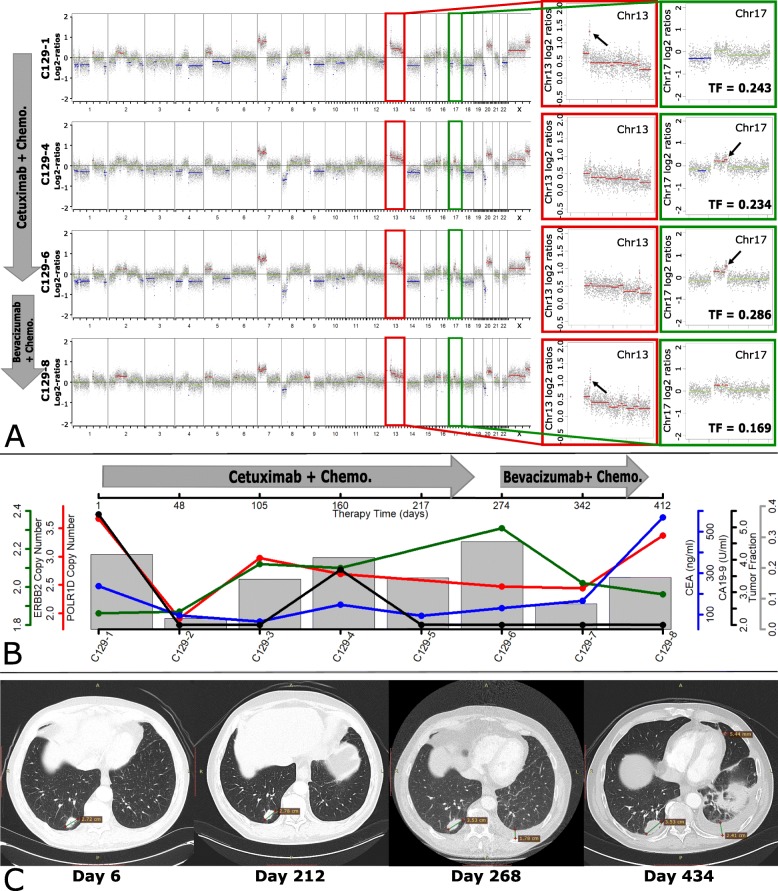
Alternating *POLR1D* and *ERBB2* amplifications in serial plasma analyses of patient C129. **a** Genome-wide log2 ratio plots of plasma samples from C129 obtained before treatment with cetuximab (first), 160 days (second) after cetuximab, before bevacizumab (third), and 138 days (fourth) after bevacizumab. The insets illustrate the respective tumor fraction (TF) for each analysis and enlarged log2 ratio plots of chromosome 13 and 17, the first and the last sample showing gain of chromosome 13, with the highest copy number on chr13q12.2, the region harboring *POLR1D*. The middle 2 samples show a gain of chromosome 17 with the highest copy number on chr17q12, harboring *ERBB2*. The copy number color code is as in Fig. 4. **b** Plot illustrating blood collection time points. Red line: *POLR1D* copy number changes measured by dPCR. A decrease in *POLR1D* copy number was detected until day 274 during cetuximab treatment. After switching to bevacizumab, *POLR1D* copy number increased back within 138 days. Green line: *ERBB2* copy number changes (dPCR). *ERBB2* copy number increased until day 274 (during cetuximab treatment). After switching to bevacizumab, *ERBB2* copy number decreased back within 138 days. Blue line: CEA levels decreased in the first 3 samples and slightly increased in the fourth sample. After a slight decrease in the fifth sample, CEA continuously increased up until the last sample. Black line: CA 19-9 remained at low levels across all samples. Gray bar: tumor fraction estimated with ichorCNA. **c** Four CT images obtained on day 6 and day 212 of cetuximab treatment, before bevacizumab treatment (day 268), and 160 days after bevacizumab treatment (day 434). No significant changes were identified on day 212, consistent with stable disease. On day 268, the pre-present lung metastasis lesion became larger in the right lung and pleural effusion appeared in the left lung, indicating progressive disease. On day 434, this pre-present lesion became larger and new metastasis lesions appeared. Pleural effusion increased, and progressive disease was designated

In patient C129, we noted a 13q12.2 amplification in the first plasma sample, which disappeared after anti-EGFR treatment. According to the blood CEA values and CT scan, the tumor acquired resistance to anti-EGFR treatment within 9 months and plasma-Seq revealed a novel focal amplification on 17q12, including *ERBB2*, which represents an established mechanism of resistance to anti-EGFR therapy (Fig. 5; Additional file 3: Table S2; Additional file 2: Figure S6) [36, 60–62]. After switching to anti-VEGF treatment for 5 months, the amplification in 13q12.2 appeared again, along with an increase in CEA level and the size of the lung metastasis region (Fig. 5; Additional file 3: Table S2; Additional file 2: Figure S6). In order to confirm this apparent clonal switch between *POLR1D* and *ERBB2*, digital PCR was performed to validate copy number amplification in all involved samples (Fig. 5b). These cases suggest that amplification of 13q12.2 along with increased expression of POLR1D and subsequent increased expression of VEGFA may contribute to anti-VEGF treatment resistance.

In summary, our results suggest that *POLR1D* may act as a potential driver gene in the 13q12.2 amplification and may affect cancer progression by increasing the expression of VEGFA and EREG. Because of the higher expression of VEGFA, amplification of 13q12.2 may be involved in the acquired resistance of anti-VEGF treatment.

## Discussion

The identification of predictive biomarkers is necessary for the implementation of individualized tumor treatment in patients with cancer. Establishment of such biomarkers would allow the stratification of patients into appropriate treatment groups and help facilitate a transition from a one-size-fits-all approach to that of precision medicine. Although a considerable number of patients with mCRC will experience progression and eventually exhaust standard therapies approved for CRC, many of these patients remain candidates for further treatment strategies if they demonstrate an adequate performance score and lack significant comorbidities. As the instability of tumor genomes has been well-established, there have been tremendous efforts to track genetic tumor markers over time rather than solely at the point of diagnosis, which can in turn provide support for determining novel evidence-based therapies for these patients. Liquid biopsy has been at the forefront of such non-invasive profiling of the tumor at regular intervals throughout a patient’s treatment and has allowed for the monitoring of the evolution of the tumor genome [7–10].

Another application utilizing plasma DNA is the much-needed identification of novel driver genes. Although the 13q12.2 amplification is relatively frequent in CRC, a driver gene has not been identified in this amplification yet. The 13q12.2 region harbors the fms-like tyrosine kinase 3 (*FLT3*), a known oncogene that encodes for a tyrosine kinase which activates proliferation and differentiation pathways in hematopoietic stem cells. Although mutations in *FLT3* have been well-documented in cases of acute myeloid leukemia (AML) and other hematological malignancies [63], there is currently no substantial evidence demonstrating that *FLT3* plays a role in the tumorigenesis of CRC. One case report focused on a particular patient with amplification of *FLT3* who demonstrated response to sorafenib, an anti-FLT3 compound [64]. However, our analysis showed no correlation between *FLT3* copy number and gene expression in both the TCGA cohort and the CCLE cell line database. Furthermore, gene expression inference from TSS nucleosome positioning suggested that in our two clinical case studies C129 and C216, *FLT3* was not expressed within the 13q12.2 amplicon. Moreover, our stable FLT3-overexpressing cell line did not exhibit any significant proliferation advantage, further questioning the role of *FLT3* as an oncogene in mCRC. Our findings are in agreement with a previous study, which showed that *FLT3* amplification does not seem to be an actionable target or a proper biomarker for FLT3 inhibitors like regorafenib or sorafenib [65].

Furthermore, two studies previously published about the potential driver gene in 13q12.2 showed varied results [66, 67]. One study suggested both *LNX2* and *POLR1D* as potential driver genes in 13q12.2. However, in this particular in vitro model, the SW480 (whole chr13 gain) and DLD1 (whole chr13 balanced) cell lines were used, which thus excluded cell lines harboring amplification of 13q12 [66]. Another study which did use cell lines harboring 13q12.2 amplification demonstrated high expression of *CDX2* and therefore concluded that *CDX2* acts as a driver gene in this region [67]. According to the CCLE data, however, *CDX2* copy number is poorly correlated with gene expression, and furthermore, in a real patient dataset (TCGA), mRNA expression was not significantly different between patients harboring a gain or amplification of chr13q12.2. However, as a recent study described that *CDX2* loss through demethylation and HDAC inhibition is an adverse prognostic factor and linked to molecular features of the serrated pathway [68], *CDX2* may act as an oncogene in tumors with a high expression of *CDX2*, but this is not necessarily applicable to all cases of CRC with chr13q12.2 aberration.

Our study suggests *POLR1D* as a potential oncogene in the 13q12.2 amplification and as a novel resistance mechanism against the anti-VEGF monoclonal antibody bevacizumab (Avastin). These conclusions are based on several observations. First, *POLR1D* overexpression caused proliferation of CRC cells as demonstrated by transient siRNA knockdown in the two CRC cell lines HT29 and SW480. Second, *POLR1D* was indeed expressed in our profoundly investigated cases C129 and C216, as inferred from plasma nucleosome positioning mapping. Third, an important consequence of *POLR1D* overexpression was upregulation of *VEGFA*, as evidenced by both our in vitro experiments and the TCGA RNA-seq data. *VEGFA* is an important regulator of angiogenesis, the target of bevacizumab, and plays a role in the development, progression, and metastasis of CRC [69]. Finally, given the specificity of bevacizumab to the VEGFA ligand [54] and as recent publications showed that bevacizumab treatment induces autocrine VEGF signaling [55, 70], we investigated the occurrence of *POLR1D* amplifications in patients receiving bevacizumab to provide in vivo data. In fact, serial plasma DNA analyses revealed that in two of our patients, the 13q12.2 amplification evolved under treatment and was in both cases linked to progressive disease (Figs. 4 and 5). It will be interesting to test the relationship between the 13q12.2 amplification and bevacizumab treatment in larger patient cohorts.

 Another interesting gene is *EREG*, which encodes epiregulin, a member of the epidermal growth factor (EGF) family, which can bind to and activate EGFR and ERBB4 [56]. Higher EREG expression is considered to be a sign of higher activation of the EGFR pathway, which, in turn, means a better response to anti-EGFR treatment [71] but more resistance to drugs which target the other ERBB family members, e.g., *ERBB2* [57, 58]. The other five genes (i.e. *PPP1R15A*, *MOSPD2*, *FAM84B*, *GARS*, *KIF21B*) have demonstrated involvement in the progression of various cancers. For example, *FAM84B*, which encodes family with sequence similarity 84, member B protein, was reported to be related to the progression of prostate cancer and esophageal squamous cell carcinoma [72, 73]. *GARS* encodes glycyl-tRNA synthetase and has been shown to be involved in neddylation, a post-translational modification that controls cell cycle and proliferation and thus may play a role in cancer progression [74]. *KIF21B* encodes a member of the kinesin superfamily and was reported to be significantly associated with poor prognosis of prostate cancer patients [75]. Mutation of *PPP1R15A*, which encodes protein phosphatase 1 regulatory subunit 15A, has been shown to be a valuable biomarker for mCRC patients sensitive to bevacizumab regimens [76]. *MOSPD2* encodes motile sperm domain-containing protein 2 and has recently been reported to promote the metastasis of breast cancer [77].

Limitations of our study include low patient number and the need of a tumor content higher than 5–10% in plasma in order to conduct reliable copy number analyses. The two patient cases C129 and C216 demonstrate the potential of plasma-Seq for therapeutic monitoring; however, such analyses depend on increased and similar tumor fractions in serial plasma samples (Figs. 4 and 5). Reduced tumor content lowers the sensitivity of SCNA detection, and in order to avoid 13q amplicon false-negatives, we established the tumor fraction with the ichorCNA algorithm and excluded plasma DNA samples where the tumor fraction was too low. Another limitation of this study is that we only focused on one SCNA event. However, other somatic alterations may modulate therapeutic response to anti-VEGF treatment as well. For example, deletion of 18q11.2-q12.1, which co-occurred in 10 of our 14 chr13q12.2 amplified patients, was recently reported to be a predictive marker of survival for mCRC patients under undergoing treatment with bevacizumab [78].

This suggests that a variety of somatic alterations may govern response to anti-VEGF therapy so that further investigations are warranted.

## Conclusions

Our results suggest that monitoring somatic focal events may allow identification of driver genes in mCRC, which has meaningful implications for the identification of novel driver genes associated with late-stage cancers. 13q12.2 is frequently amplified in CRC and may be related to tumor stage and metastasis. Here, *POLR1D*, a subunit of RNA polymerases I and III, was established as the most likely driver gene in this frequently amplified region, which may play a role in the oncogenesis of CRC by affecting *VEGFA* and *EREG* expression. As this may result in the acquired resistance to bevacizumab, *POLR1D* is a potential therapeutic target for mCRC.

## Supplementary information

**Additional file 1.** Age spectrum and sex distribution of the CRC cohort used in this study.

**Additional file 2: Figure S1.** Identification of the 13q amplicon. **Figure S2.** Genome-wide SCNA plots of all 13q12 focal amplification patients and three CRC cell lines used in this study. **Figure S3.** Definition of the minimally 13q12 amplified region and identification of the potential driver genes. **Figure S4.** Expression analyses for identification of the potential driver gene in the 13q12.2 amplicon. **Figure S5.** Emergence of the 13q12 amplicon under bevacizumab treatment in patient C216. **Figure S6.** Alternating POLR1D and ERBB2 amplifications in serial plasma analyses of patient C129.

**Additional file 3: Table S1.** Summary of genes differently expressed after POLR1D knockdown in both HT29 and SW480 cells and their expression levels in TCGA dataset. **Table S2.** Summary of all patient data for C216 and C129. **Table S3.** Summary of all the siRNA oligos.

**Additional file 4: **Summary of recurrent focal events in our cohort and TCGA dataset. **Our Cohort**: Summary of all recurrent focal events with a frequency over 0.01 (Upper) or 0.05 (Bottom) in our CRC cohort. Potential driver genes were identified as in Fig. 1a. **TCGA**: Summary of all recurrent focal events with a frequency over 0.01 (Upper) or 0.05 (Bottom) in TCGA cohort. Potential driver genes were identified as in Fig. 1a.

## Data Availability

The datasets and computer code used in this study are available in the following databases: • RNA-seq data: Gene Expression Omnibus GSE140198 (https://www.ncbi.nlm.nih.gov/geo/query/acc.cgi?acc= GSE140198) • Low coverage WGS data: European Genome-phenome Archive EGAS00001003791 (https://www.ebi.ac.uk/ega/studies/EGAS00001003791) • Focal SCNA identification analysis in R: GitHub (https://github.com/PeterUlz/FocalAmplifications/tree/master/Focal_amplifications_in_R.ipynb) • TCGA-COADREAD RNA-seq: Broad GDAC Firehose [40] illuminahiseq_rnaseqv2RSEM_genes_normalized (http://gdac.broadinstitute.org/) • TCGA-COADREAD clinic data: Broad GDAC Firehose [40] Clinical_Pick_Tier1 (http://gdac.broadinstitute.org/) • TCGA copy number data: NCI Genomic Data Commons [41] ABSOLUTE-annotated seg file (https://gdc.cancer.gov/about-data/publications/pancanatlas)

## Abbreviations

AML: Acute myeloid leukemia
CCLE: Cancer Cell Line Encyclopedia
CRC: Colorectal cancer
CT: Computerized tomography
dPCR: Digital PCR
EGF: Epidermal growth factor
EGFR: Epidermal growth factor receptor
ERBB4: Receptor tyrosine-protein kinase erbB-4
FDA: Food and Drug Administration
FFPE: Formalin-fixed paraffin-embedded
FLT3: Fms-like tyrosine kinase 3
GISTIC: Genomic Identification of Significant Targets in Cancer
mCRC: Metastatic colorectal cancer
rRNA: Ribosomal ribonucleic acid
SCNA: Somatic copy number alteration
SVM: Support vector machine
TCGA: The Cancer Genome Atlas
TSS: Transcription start site
VEGFA: Vascular endothelial growth factor A
