# Supplementary material for: Cell-free DNA analysis reveals POLR1D-mediated resistance to bevacizumab in colorectal cancer
Source: Genome Med. 2020 Feb 22;12:20. doi: 10.1186/s13073-020-0719-6 (PMC7036260; doi:10.1186/s13073-020-0719-6)
Supplement: Supplementary file 2 — Additional file 2: Figure S1. Identification of the 13q amplicon. Figure S2. Genome-wide SCNA plots of all 13q12 focal amplification patients and three CRC cell lines used in this study. Figure S3. Definition of the minimally 13q12 amplified region and identification of the potential driver genes. Figure S4. Expression analyses for identification of the potential driver gene in the 13q12.2 amplicon. Figure S5. Emergence of the 13q12 amplicon under bevacizumab treatment in patient C216. Figure S6. Alternating POLR1D and ERBB2 amplifications in serial plasma analyses of patient C129. [file 13073_2020_719_MOESM2_ESM.docx]

**Supplementary Figure Legends**

**Figure S1:** **Identification of the 13q amplicon.** (A) Circos plot showing the recurrent focal events present in our mCRC cohort in comparison to the TCGA cohort. The three regions including 12p12.1, 13q12.13-q12.3 and 8p11.23-p11.22 were present in more than 5% of our cohort. Overlap between the two cohorts was seen on chr8 and 13, whereas our cohort had a higher percentage of 12p12.1 events when compared to the TCGA database, most likely the result of a higher frequency of patients treated with anti-EGFR therapy in our cohort. (B) Comparison of SCNA profiles in patients with available primary tumor (PT) tissue and ctDNA samples with corresponding tumor fraction (TF) as estimated by ichorCNA. Four patients (C123, C109, C178 and C74) already had detectable 13q12 focal amplification in the primary tumor, whereas this event was acquired at a later time point in the ctDNA samples of five patients (C240, C118, C79, C206 and C166). Primary tumor tissue was unavailable for analysis in four patients (C95, C110, C112, and C129).

**Figure S2: Genome-wide SCNA plots of all 13q12 focal amplification patients and three CRC cell lines used in this study.** Oxco2 cells (bottom left) do not harbor any changes on chr13 whereas HT29 (bottom right) harbors focal amplification of chr13 and SW480 (bottom right) exhibits a complete gain of chr13.

**Figure S3: Definition of the minimally 13q12 amplified region and identification of the potential driver genes.** (A) Plots demonstrating the process of defining the minimally 13q12 amplified region (top) and involved genes (bottom), illustrating the overlapping ranges between our cohort, the TCGA dataset and GISTIC data as well as the overlapping broad and focal peaks. (B) Box plots showing lack of significant correlation between copy number and mRNA expression (log10(normalized RSEM value + 1)) in the TCGA data of three genes *FLT3*, *GSX1* and *PRHOXNB* (Control/matched normal tissue: n=51; Balanced: n=196; Gain: n=129; Amplification: n=46). (C) Scatter plots illustrating lack of significant correlation between copy number and mRNA expression (log2 (TPM + 1)) in 58 CRC cell lines from the CCLE database for three genes (*FLT3*, *GSX1* and *PRHOXNB*). R values and P values were calculated by using Pearson correlation test. The red line represent for the noise threshold. (TPM = 1)

**Figure S4:** **Expression analyses for identification of the potential driver gene in the 13q12.2 amplicon.** (A) Silencing of *CDX2, LNX2, PAN3, PDX1 and POLR1D* with 2 different siRNA constructs. RT-PCR showing that silencing provided sufficient knockdown of gene expression in both cell lines HT29 and SW480. (B) Box plots illustrating the differential expression (normalized DESeq2 read count) of *FAM84B*, *GARS*, *KIF21B*, *MOSPD2*, *PPP1R15A* and *POLR1D* between negative control (SCR, scrambled siRNA) and *POLR1D* knockdown in SW480 (SCR: n=6; siPOLR1D2: n=3; siPOLR1D3: n=3) and HT29 (SCR: n=4; siPOLR1D2: n=2; siPOLR1D3: n=2) cell lines. Expression of *FAM84B*, *GARS*, *KIF21B*, *MOSPD2*, *PPP1R15A* and *POLR1D* was suppressed after *POLR1D* silencing. Adjusted P values were calculated by DESeq2, an R package. (C) Violin plots showing a significant increase *FAM84B*, *GARS*, *KIF21B*, *MOSPD2*, *PPP1R15A*, *VEGFA*, *EREG* and *POLR1D* expression (normalized RSEM value) in the chr13q12.2 aberrant cohort (n=175) compared with the balanced group (n=196).

**Figure S5: Emergence of the 13q12 amplicon under bevacizumab treatment in patient C216.** (Left panel) Genome-wide log2-ratio plots of plasma samples from C216 while undergoing bevacizumab treatment. The inset illustrates enlarged log2-ratio plots of chromosome 13, which demonstrated a balanced region up until sample C216-10, which harbored a slight gain and ultimately increased into a focal amplification with sample C216-12. Copy number gains are shown in red and copy number losses in blue. (Right panel) 5 CT images obtained at 5 different time points best corresponding to the blood draws. Scans from Day 10-Day 152 did not demonstrate any significant changes in the lesion, representing stable disease, whereas comparison of the scans from Day 152-Day 222 demonstrated significant increase in the target lesion diameter, reaching its largest size at 5.70cm on Day 276, which coincided with the presence of the focal amplification in plasma sample C216-12.

**Figure S6: Alternating POLR1D and ERBB2 amplifications in serial plasma analyses of patient C129.** (Left panel) Genome-wide log2-ratio plots of all plasma samples from C129 obtained during and after cetuximab treatment (C129-1 to C129-6) as well as before and after bevacizumab treatment (C129-6 to C129-8). The inset illustrates enlarged log2-ratio plots of chromosome 13 and 17, the first and the last sample showing gain of chromosome 13 with the highest copy number gain on chr13 q12.2, the region that harbors the *POLR1D* gene. The middle 2 samples show gain of chromosome 17 with the highest copy number gain on chr17 q12, the location of the *ERBB2* gene. Copy number gains are depicted in red and copy number losses in blue. (Right panel) 7 CT images obtained at 7 different time points best corresponding to the blood draws. Stable disease was demonstrated between samples C129-1 and C129-6, with the target lung metastasis lesion not showing any changes. However, on Day 268, the patient exhibited progressive disease with an increase in the lung target lesion on the right side as well as the development of pleural effusion in the left lung. On Day 434, the target lung lesion became even larger and was accompanied by the appearance of new metastatic lesions as well as an increase in the pleural effusion, indicating once again progressive disease.

**Figure S1:** **Identification of the 13q amplicon.**

**
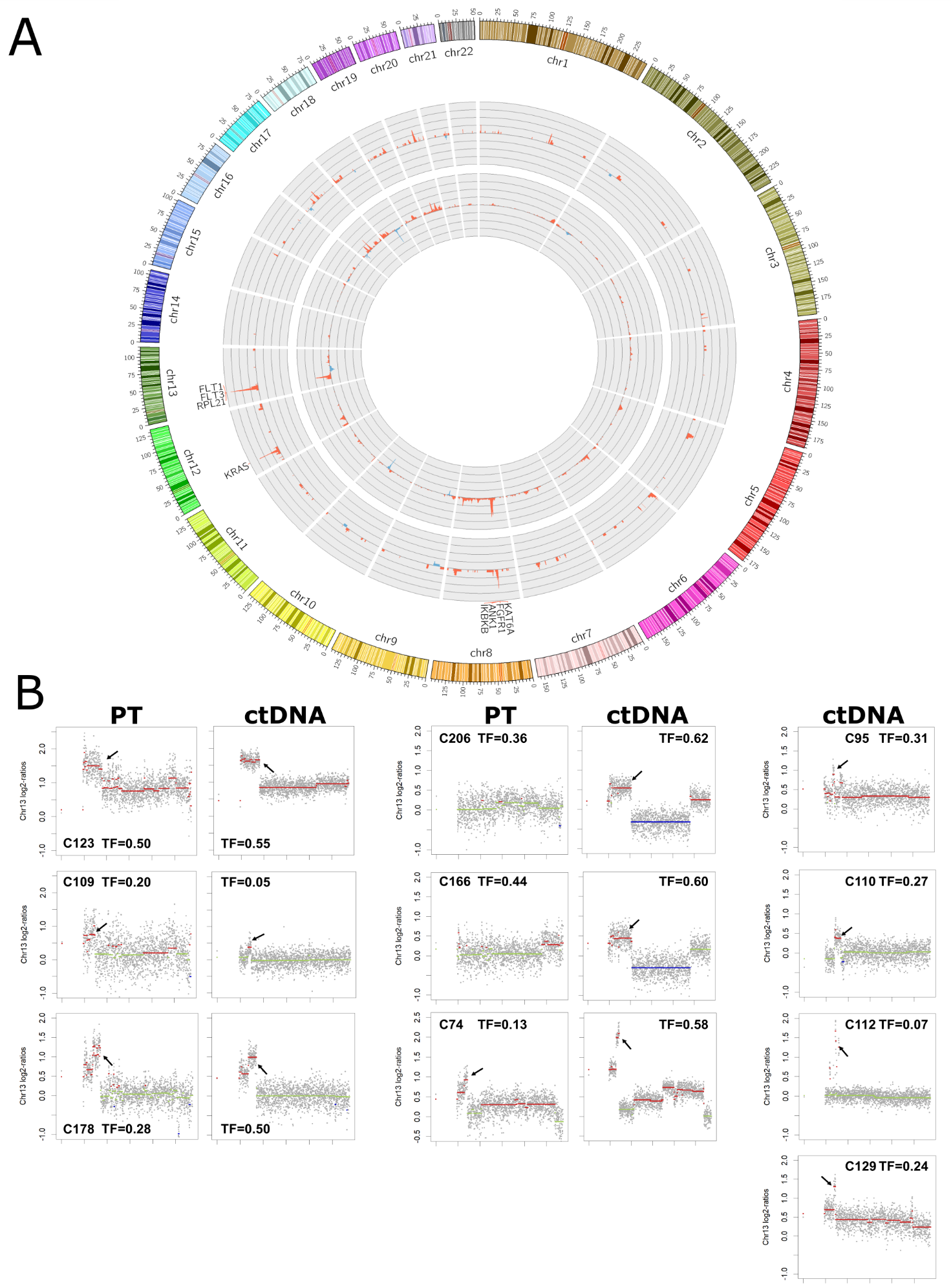
**

**Figure S2: Genome-wide SCNA plots of all 13q12 focal amplification patients and three CRC cell lines used in this study.**

**
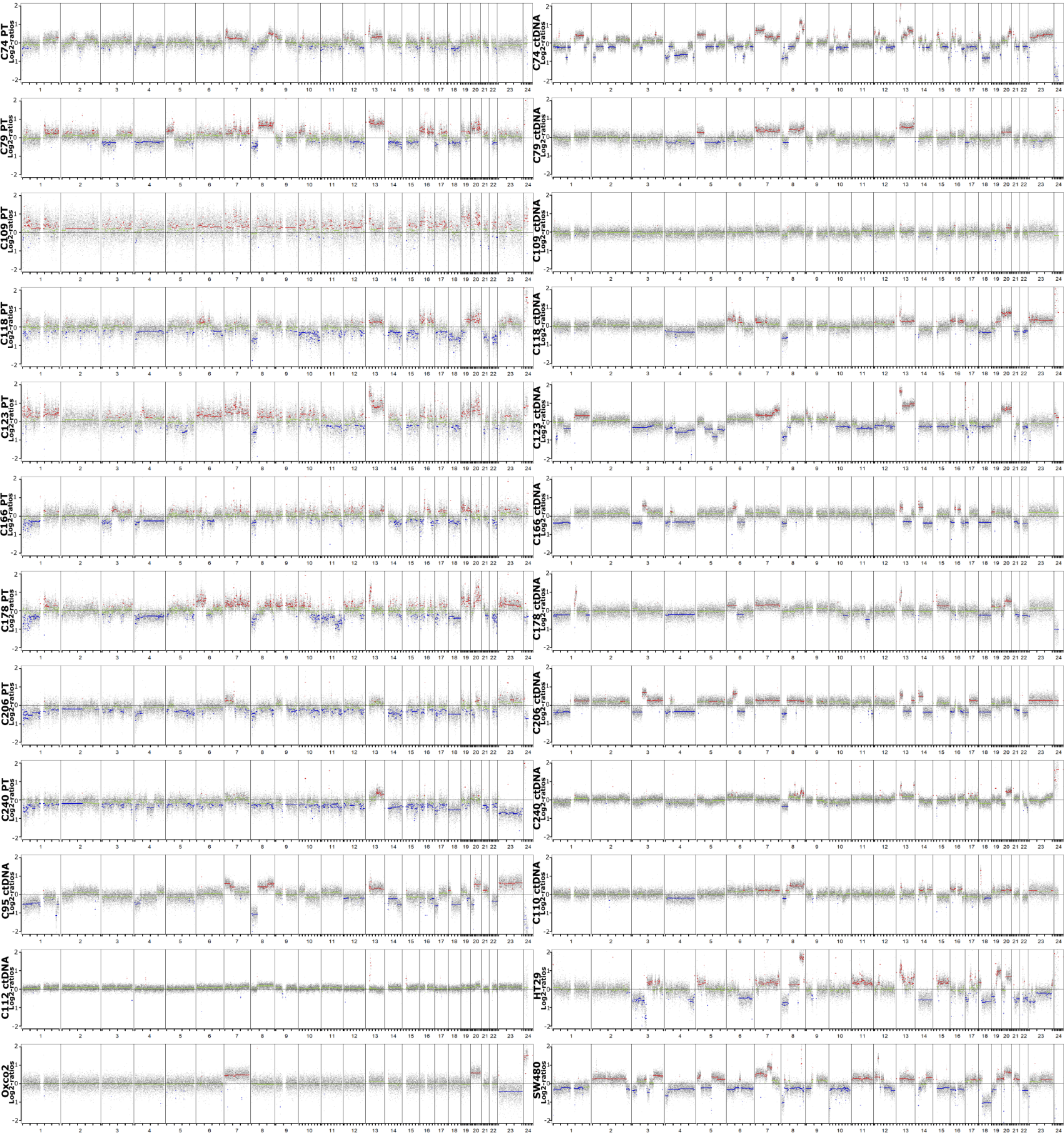
**

**Figure S3: Definition of the minimally 13q12 amplified region and identification of the potential driver genes.**

**
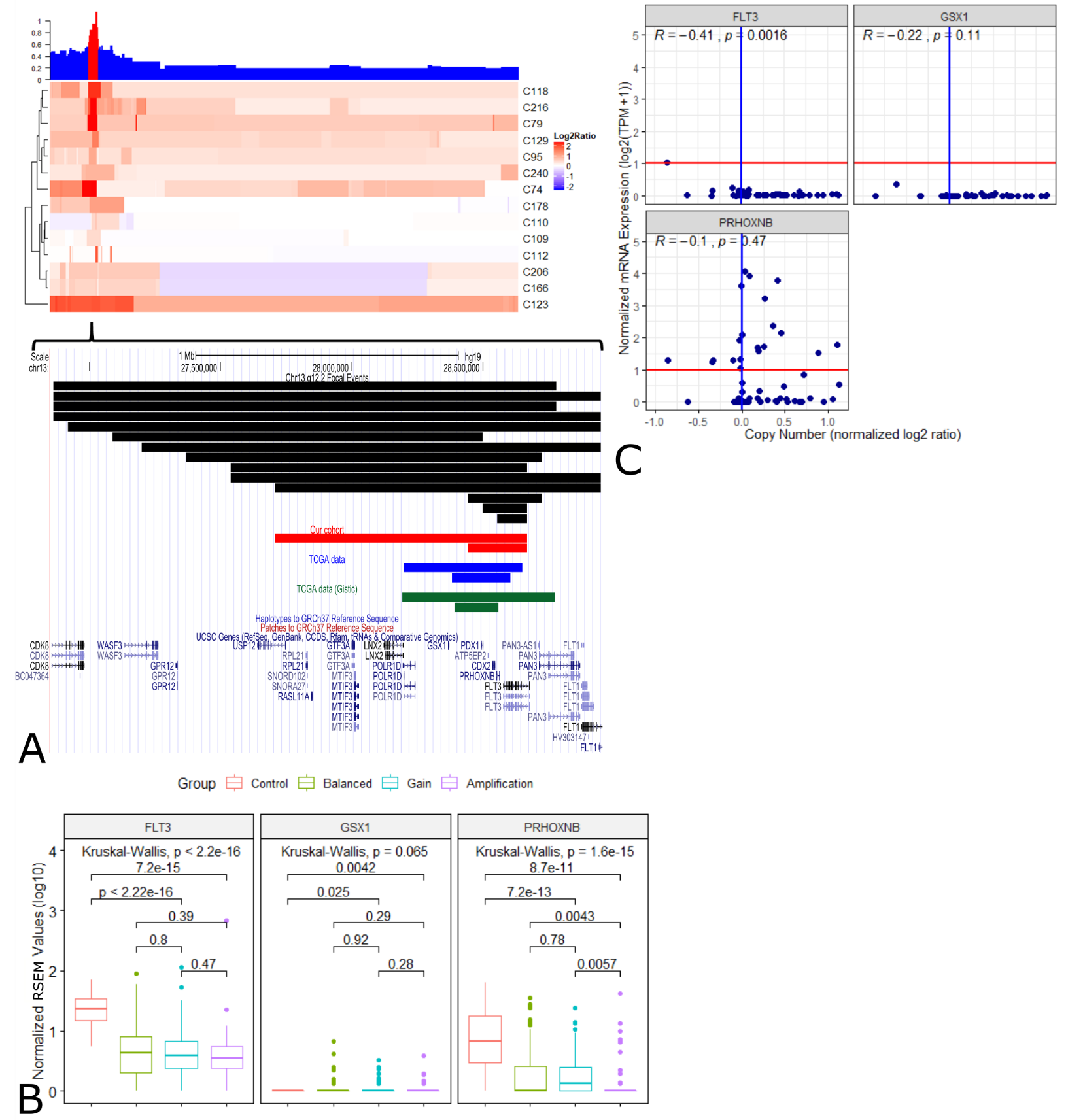
**

**Figure S4:** **Expression analyses for identification of the potential driver gene in the 13q12.2 amplicon.**

**
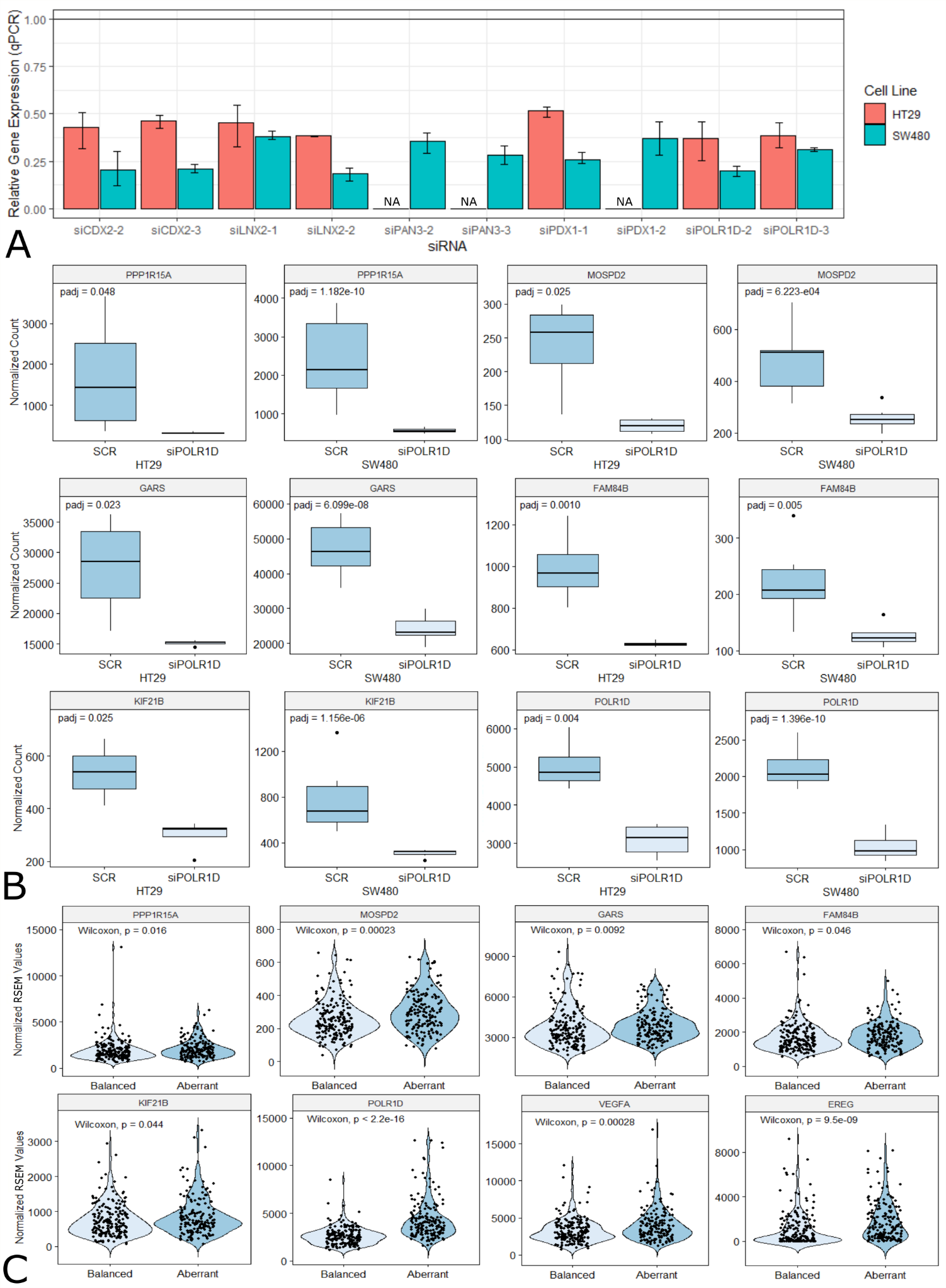
**

**Figure S5: Emergence of the 13q12 amplicon under bevacizumab treatment in patient C216.**

**
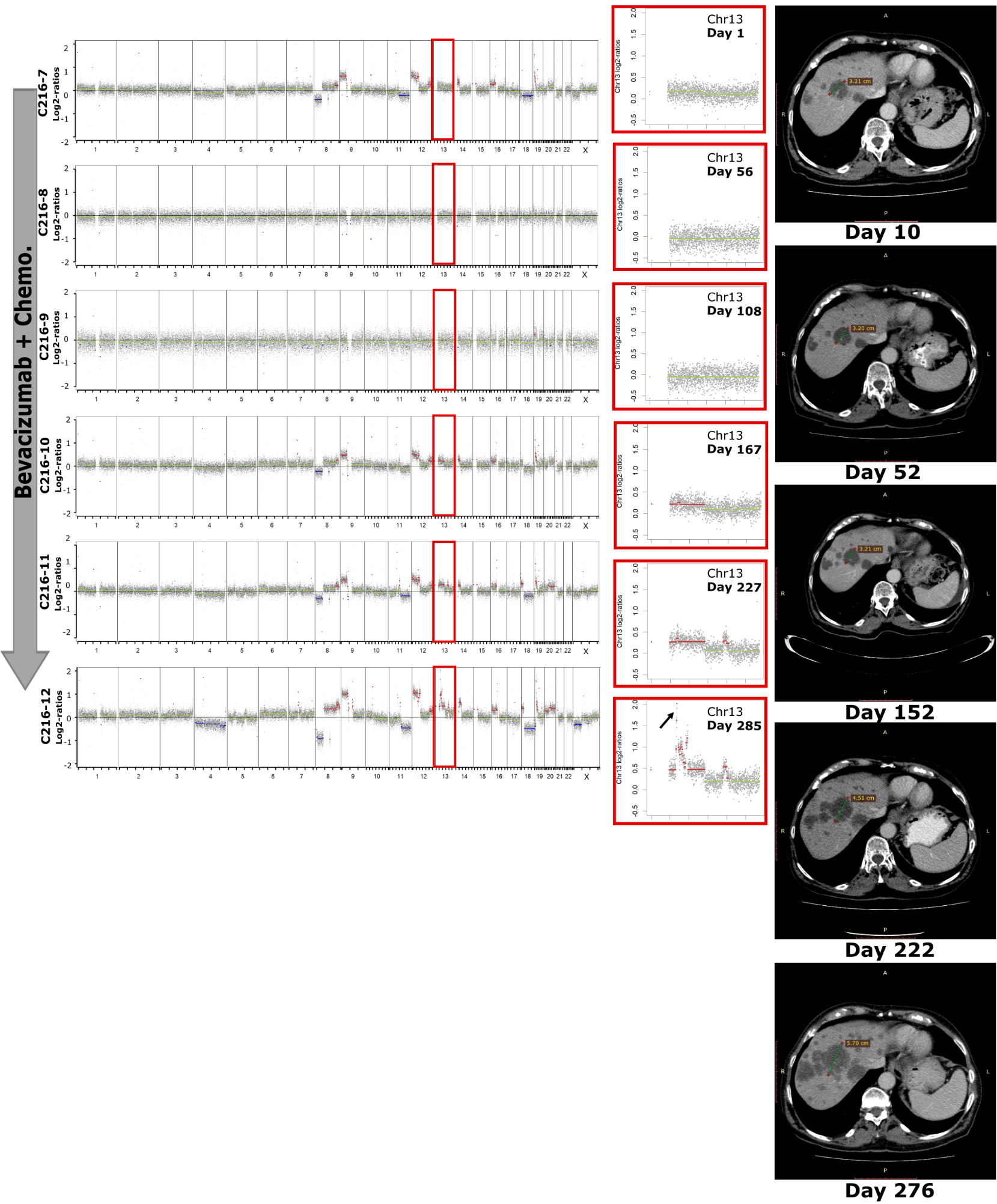
**

**Figure S6: Alternating POLR1D and ERBB2 amplifications in serial plasma analyses of patient C129.**

**
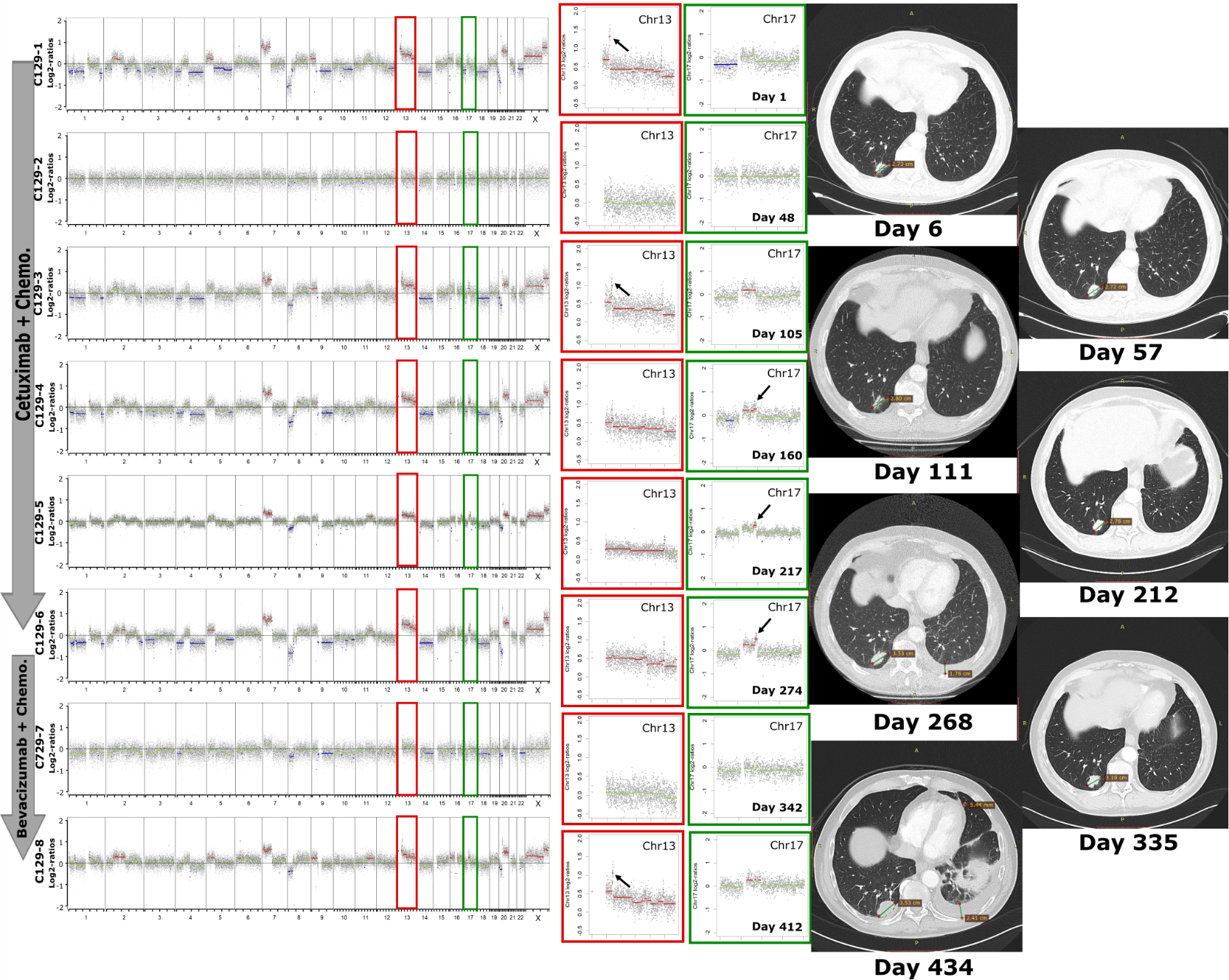
**
