# Supplementary material for: Cell-free DNA analysis reveals POLR1D-mediated resistance to bevacizumab in colorectal cancer
Source: Genome Med. 2020 Feb 22;12:20. doi: 10.1186/s13073-020-0719-6 (PMC7036260; doi:10.1186/s13073-020-0719-6)
Supplement: Supplementary file 3 — Additional file 3: Table S1. Summary of genes differently expressed after POLR1D knockdown in both HT29 and SW480 cells and their expression levels in TCGA dataset. Table S2. Summary of all patient data for C216 and C129. Table S3. Summary of all the siRNA oligos. [file 13073_2020_719_MOESM3_ESM.docx]

**Supplementary Table Legends:**

**Table S1:** **Summary of genes differently expressed after POLR1D knockdown in both HT29 and SW480 cells and their expression levels in TCGA dataset.** 45 genes showed consistent expression change in HT29 and SW480 cell lines after POLR1D knockdown, log2 fold change and P-value are listed in the table. Additionally, expression levels of these genes were compared between 13q12.2 balanced and aberrant groups in the TCGA dataset. (Log2FC: log2 fold change of gene expression (Log2 (siPOLR1D/SCR)). *: adjusted P-value calculated by DESeq2 package. +: P-value calculated using Wilcoxon test.)

**Table S2: Summary of all patient data for C216 and C129**. Therapy, CEA, CA199 values and CT evaluation results of each blood collection time point of C216 and C129 are summarized below together with digital PCR results (copy number of POLR1D and ERBB2) and tumor fraction estimation. (CN: copy number detected by dPCR; TF: Tumor fraction calculated by ichorCNA)

**Table S3: Summary of all the siRNA oligos.** siRNA oligos used in this study and their transfection efficiency in HT29 and SW480 cell lines, respectively.

**Table S1: Summary of genes differently expressed after POLR1D knockdown in both HT29 and SW480 cells and their expression levels in TCGA dataset.**

| **Gene** | **HT29 Log2FC** | **HT29 Padj*** | **SW480 Log2FC** | **SW480 Padj*** | **TCGA**  **P value+** | **TCGA Balanced Median** | **TCGA Aberrant Median** |
| --- | --- | --- | --- | --- | --- | --- | --- |
| POLR1D | -0.7128229 | 0.00449661 | -1.0301794 | 1.40E-10 | 4.99054E-26 | 2513.89995 | 3793.959 |
| EREG | -1.4452929 | 0.0293995 | -2.0894708 | 5.23E-05 | 9.54296E-09 | 373.00665 | 1301.689 |
| VEGFA | -1.6946917 | 0.02176345 | -1.1063407 | 0.0004098 | 0.000279851 | 2938.6879 | 3403.325 |
| GARS | -0.8629731 | 0.0233373 | -0.9643462 | 6.10E-08 | 0.009231291 | 3417.0136 | 3774.436 |
| FAM84B | -0.659154 | 0.00968512 | -0.804392 | 0.00455962 | 0.04643275 | 1522.4487 | 1709.143 |
| PPP1R15A | -2.4018937 | 0.04823467 | -2.0948666 | 1.18E-10 | 0.01555854 | 1667.57915 | 1845.758 |
| KIF21B | -0.8513724 | 0.02504416 | -1.3588441 | 1.16E-06 | 0.04393476 | 644.7362 | 710.1464 |
| MOSPD2 | -0.9839675 | 0.02504416 | -0.9077483 | 0.00062231 | 0.000228129 | 238.4314 | 287.4251 |
| MUC12 | 2.188039 | 0.0233373 | 1.6257477 | 0.01996535 | 1.21902E-07 | 987.96985 | 2679.441 |
| DPEP1 | 1.0391931 | 0.03239612 | 1.0525786 | 0.01479816 | 0.000155794 | 2265.18 | 3783.097 |
| HSD11B2 | 1.2780104 | 0.00190125 | 0.9474168 | 0.00388939 | 0.006967245 | 1732.68055 | 2161.517 |
| SULT2B1 | 0.6447917 | 0.01868489 | 1.0435684 | 0.01782547 | 0.007513332 | 365.1714 | 484.6336 |
| DOCK11 | 2.0156599 | 0.02907911 | 1.0497497 | 0.0318923 | 0.00976697 | 348.3723 | 435.7683 |
| PROX1 | 1.9051311 | 0.04074343 | 0.8948489 | 0.00071571 | 0.003398216 | 163.9231 | 205.283 |
| MORN4 | -3.2213428 | 0.0007858 | -0.6895181 | 0.02557299 | 8.29056E-05 | 87.48645 | 72.8302 |
| CTH | -1.9229918 | 0.01859367 | -1.5466323 | 4.52E-06 | 0.001264022 | 122.837 | 103.0207 |
| CHAC1 | -2.3938038 | 5.73E-05 | -1.3336525 | 0.01750432 | 0.004218698 | 168.54035 | 141.1333 |
| SLFN5 | -1.768586 | 0.00524378 | -2.2540744 | 5.60E-05 | 0.001609381 | 293.0512 | 242.3706 |
| SESN2 | -1.9412111 | 0.00598858 | -2.0298841 | 8.62E-05 | 0.001005439 | 454.92015 | 391.0093 |
| SLC38A2 | -1.1251007 | 0.00175032 | -1.1995282 | 0.01106125 | 0.03182495 | 2945.4884 | 2855.136 |
| SLC7A11 | -1.6614718 | 0.00196317 | -1.678202 | 0.00072969 | 0.000110188 | 488.9114 | 378.706 |
| APOL6 | -1.0914653 | 0.00060994 | -1.5501895 | 0.00354982 | 1.02788E-06 | 1858.86865 | 1568.716 |
| ANXA1 | -0.6573977 | 0.03453267 | -1.9886396 | 1.25E-08 | 0.001765714 | 1445.2876 | 1114.838 |
| MTHFD2 | -1.3845903 | 0.0123018 | -0.9386858 | 0.00348556 | 2.39712E-08 | 2013.44365 | 1544.529 |
| XBP1 | -0.9617556 | 0.02530649 | -0.9092207 | 7.07E-05 | 1.01505E-05 | 5308.0528 | 4634.824 |
| DDIT4 | -2.2934595 | 5.73E-05 | -1.6754201 | 0.00909115 | 0.000306201 | 3003.3448 | 2195.472 |
| TUBB | 1.208217 | 0.02504902 | 1.7314544 | 0.02410687 | 0.05132316 | 16494.4767 | 15594.8 |
| ULBP1 | -2.3746428 | 0.00293501 | -1.4778163 | 0.0048564 | 0.06732695 | 16.90435 | 13.9894 |
| BEST1 | -1.6286437 | 0.04111525 | -1.581836 | 0.00190779 | 0.07428151 | 20.07455 | 24.1673 |
| LONRF1 | -2.3994092 | 0.00255374 | -0.6202471 | 0.03162924 | 0.1227335 | 291.9928 | 317.0184 |
| SP5 | 2.4616033 | 0.01598578 | 0.704191 | 0.00226095 | 0.1627161 | 189.3337 | 132.5616 |
| HSPA13 | -1.5490545 | 0.03706914 | -1.0112029 | 1.75E-05 | 0.1939381 | 600.5065 | 568.3804 |
| IL3RA | 1.500118 | 0.0233373 | 1.4207262 | 0.01876354 | 0.2459109 | 111.17865 | 135.8867 |
| ATF4 | -0.7287908 | 0.01288611 | -0.7945738 | 4.41E-05 | 0.4245156 | 6539.15705 | 6439.585 |
| STRC | 2.1308239 | 0.00293501 | 1.3832091 | 0.01774951 | 0.4389994 | 5.28165 | 5.5633 |
| GDF15 | -2.5897728 | 0.04074343 | -1.6993912 | 0.0022295 | 0.5149138 | 2799.57955 | 2431.004 |
| GPX8 | -1.6006957 | 0.00011944 | -1.1492875 | 0.00342902 | 0.5306847 | 307.09955 | 298.0921 |
| SLC1A4 | -2.2725932 | 0.00074873 | -0.9699159 | 3.38E-06 | 0.5551172 | 1261.316 | 1267.928 |
| INSIG2 | -0.6693009 | 0.0142507 | -0.8393069 | 0.00472022 | 0.5708263 | 491.41485 | 500.7547 |
| GPT2 | -0.9519746 | 0.02176345 | -0.9747738 | 2.99E-06 | 0.6544763 | 1572.77765 | 1526.729 |
| LGALS1 | -0.799721 | 0.02530649 | -0.744645 | 0.00746905 | 0.6621949 | 2338.41245 | 2124.263 |
| AARS | -1.4168308 | 0.04716745 | -0.8610822 | 0.00056494 | 0.7566797 | 3537.6686 | 3404.814 |
| ATF3 | -3.618937 | 0.00568068 | -2.114485 | 0.00057144 | 0.7566797 | 773.1627 | 785.7408 |
| CLIP4 | -1.6842405 | 0.04890417 | -1.3437026 | 0.00056158 | 0.7603699 | 46.61535 | 49.3107 |
| DDIT3 | -3.4201171 | 0.01604729 | -1.9581415 | 4.04E-05 | 0.9748566 | 356.5413 | 369.2023 |

**Table S2: Summary of all the data of patient C216 and C129**.

| **Sample** | **Treatment** | **Plasma collection** | **POLR1D CN*** | **ERBB2**  **CN*** | **Biomarker testing** | **CEA (ng/ml)** | **CA199 (U/ml)** | **TF** | **CT testing** | **Clinical evaluation** |
| --- | --- | --- | --- | --- | --- | --- | --- | --- | --- | --- |
| C216-7 | FOLFOX | Day1 | 1.85 | NA | Day1 | 77 | 403 | 0.331 | Day10 | Baseline |
| C216-8 | FOLFOX + BEV. | Day56 | 1.58 | NA | Day50 | 33 | 114 | 0.041 | Day52 | SD |
| C216-9 | FOLFOX + BEV. | Day108 | 1.59 | NA | Day94 | 22.4 | 206.3 | 0.042 | NA | SD |
| C216-10 | FOLFOX + BEV. | Day167 | 1.86 | NA | Day167 | 65.6 | 670 | 0.179 | Day152 | SD |
| C216-11 | 5-Fu + BEV. | Day227 | 2.21 | NA | Day227 | 146 | 901.4 | 0.213 | Day222 | PD |
| C216-12 | 5-Fu | Day285 | 10.07 | NA | Day285 | 356 | 2265 | 0.487 | Day276 | PD |
| C129-1 | No treatment | Day1 | 3.66 | 1.86 | Day1 | 237 | 5.4 | 0.243 | Day6 | Baseline |
| C129-2 | FOLFIRI + Cet. | Day48 | 1.91 | 1.87 | Day48 | 96.1 | 2 | 0.036 | Day57 | SD |
| C129-3 | FOLFIRI + Cet. | Day105 | 2.97 | 2.12 | Day105 | 66.6 | 2 | 0.163 | Day111 | SD |
| C129-4 | FOLFIRI + Cet. | Day160 | 2.69 | 2.10 | Day160 | 146.8 | 3.7 | 0.234 | NA | SD |
| C129-5 | FOLFIRI + Cet. | Day217 | NA | NA | Day203 | 94.2 | 2 | 0.167 | Day212 | SD |
| C129-6 | FOLFOX + BEV. | Day274 | 2.47 | 2.31 | Day268 | 130 | 2 | 0.286 | Day268 | PD/Baseline |
| C129-7 | FOLFOX + BEV. | Day342 | 2.44 | 2.02 | Day342 | 167.4 | 2 | 0.084 | Day335 | SD |
| C129-8 | No treatment | Day412 | 3.37 | 1.96 | Day440 | 567.7 | 2 | 0.169 | Day434 | PD |

**Table S3: Summary of all the siRNA oligos.**

| **siRNA** | **Life-Tech ID** | **Target gene** | **Knockdown efficiency in HT29** | **Knockdown efficiency in SW480** |
| --- | --- | --- | --- | --- |
| siCDX2-2 | s2876 | CDX2 | Successful | Successful |
| siCDX2-3 | s2878 | CDX2 | Successful | Successful |
| siLNX2-1 | s48196 | LNX2 | Successful | Successful |
| siLNX2-2 | s48195 | LNX2 | Successful | Successful |
| siPAN3-1 | s48721 | PAN3 | Insufficient knockdown | Successful |
| siPAN3-2 | s48723 | PAN3 | Insufficient knockdown | Successful |
| siPAN3-3 | s48722 | PAN3 | Insufficient knockdown | Successful |
| siPDX1-1 | s7488 | PDX1 | Successful | Successful |
| siPDX1-2 | s223944 | PDX1 | Insufficient knockdown | Successful |
| siPOLR1D-2 | 23515 | POLR1D | Successful | Successful |
| siPOLR1D-3 | 119496 | POLR1D | Successful | Successful |
| siPOLR1D-4 | 39457 | POLR1D | Successful | Successful |
